# The Effect of Shame on Patients With Social Anxiety Disorder in Internet-Based Cognitive Behavioral Therapy: Randomized Controlled Trial

**DOI:** 10.2196/15797

**Published:** 2020-07-20

**Authors:** Haoyu Wang, Qingxue Zhao, Wenting Mu, Marcus Rodriguez, Mingyi Qian, Thomas Berger

**Affiliations:** 1 School of Psychological and Cognitive Sciences Peking University Beijing China; 2 School of Psychological and Cognitive Sciences Beijing Key Laboratory for Behavior and Mental Health Peking University Beijing China; 3 Center for the Treatment and Study of Anxiety University of Pennsylvania Pennsylvania, PA United States; 4 Department of Psychology Pitzer College California, CA United States; 5 Department of Clinical Psychology and Psychotherapy University of Bern Bern Switzerland

**Keywords:** internet-based intervention, cognitive behavioral therapy, phobia, social, shame, implosive therapy

## Abstract

**Background:**

Prior research has demonstrated the efficacy of internet-based cognitive behavioral therapy (ICBT) for social anxiety disorder (SAD). However, it is unclear how shame influences the efficacy of this treatment.

**Objective:**

This study aimed to investigate the role shame played in the ICBT treatment process for participants with SAD.

**Methods:**

A total of 104 Chinese participants (73 females; age: mean 24.92, SD 4.59 years) were randomly assigned to self-help ICBT, guided ICBT, or wait list control groups. For the guided ICBT group, half of the participants were assigned to the group at a time due to resource constraints. This led to a time difference among the three groups. Participants were assessed before and immediately after the intervention using the Social Interaction Anxiety Scale (SIAS), Social Phobia Scale (SPS), and Experience of Shame Scale (ESS).

**Results:**

Participants’ social anxiety symptoms (self-help: differences between pre- and posttreatment SIAS=−12.71; Cohen *d*=1.01; 95% CI 9.08 to 16.32; *P*<.001 and differences between pre- and posttreatment SPS=11.13; Cohen *d*=0.89; 95% CI 6.98 to 15.28; *P*<.001; guided: SIAS=19.45; Cohen *d*=1.20; 95% CI 14.67 to 24.24; *P*<.001 and SPS=13.45; Cohen *d*=0.96; 95% CI 8.26 to 18.64; *P*<.001) and shame proneness (self-help: differences between pre- and posttreatment ESS=7.34; Cohen *d*=0.75; 95% CI 3.99 to 10.69; *P*<.001 and guided: differences between pre- and posttreatment ESS=9.97; Cohen *d*=0.88; 95% CI 5.36 to 14.57; *P*<.001) in both the self-help and guided ICBT groups reduced significantly after treatment, with no significant differences between the two intervention groups. Across all the ICBT sessions, the only significant predictors of reductions in shame proneness were the average number of words participants wrote in the exposure module (β=.222; SE 0.175; t_96_=2.317; *P*=.02) and gender (β=−.33; SE 0.002; t_77_=−3.13; *P*=.002). We also found a mediation effect, wherein reductions in shame fully mediated the relationship between the average number of words participants wrote in the exposure module and reductions in social anxiety symptoms (SIAS: β=−.0049; SE 0.0016; 95% CI −0.0085 to −0.0019 and SPS: β=−.0039; SE 0.0015; 95% CI −0.0075 to −0.0012).

**Conclusions:**

The findings of this study suggest that participants’ engagement in the exposure module in ICBT alleviates social anxiety symptoms by reducing the levels of shame proneness. Our study provides a new perspective for understanding the role of shame in the treatment of social anxiety. The possible mechanisms of the mediation effect and clinical implications are discussed.

**Trial Registration:**

Chinese Clinical Trial Registry ChiCTR1900021952; http://www.chictr.org.cn/showproj.aspx?proj=36977

## Introduction

### Background

Internet-based cognitive behavioral therapy (ICBT) entails similar content to conventional in-person cognitive behavioral therapy (CBT), which has been proven to have treatment effects equivalent to pharmacological treatments [1,2]. ICBT is developing rapidly because of its convenience, low cost, and wide range of use. ICBT has shown significant and long-standing effects for various psychiatric disorders [3-5], especially social anxiety disorder (SAD) [6]. A longitudinal study showed that improvements in SAD symptoms after ICBT were maintained at 5-year follow-up [7].

Shame shares many similarities with social anxiety, including self-directed attention; fear of negative evaluations from others; and regarding oneself as unwelcome, unattractive, or worthless in others’ view [8-10]. Shame proneness, as a dispositional affective sensitivity to the emotion of shame, is a stable trait that refers to individuals’ cognitive, affective, and behavioral responses to transgressions [11,12]. Empirical and meta-analytic studies suggest that shame plays an important role in the development and maintenance of social anxiety [13,14]. Helsel’s [15] study of children’s SAD and shame experiences showed that certain degrees of shame experience could cause social anxiety. Some studies also showed significant correlations between shame proneness, SAD, social avoidance, and distress [16-18]. Several studies also showed that both individual and group CBT significantly reduce shame in patients with SAD [16,19]. However, they did not explore which part of CBT changes participants’ shame proneness. Hedman et al [20] proposed cognitive modification and exposure exercises as possible mechanisms.

Some empirical studies have investigated the relationship between shame and social anxiety through self-reported scales [13,17,18], and shame has been suggested to play an important role in SAD. In a longitudinal study, Li et al [21] verified the important influence of shame on social anxiety and found that reductions in shame proneness led to improvements in social anxiety. In addition, several studies found that CBT and compassion-focused therapy (a treatment speciﬁcally designed for people with high levels of shame) reduced patients’ body shame [22,23]. Although some studies have explored the relationship between shame and social anxiety symptoms, it is still unknown what role shame plays in the ICBT treatment of SAD.

### Objective of This Study

In this study, we investigated the following questions among a sample of Chinese individuals with SAD: (1) Is shame proneness significantly reduced over the course of treatment using a Chinese version of the ICBT? and (2) If so, which modules in the ICBT influence the levels of shame proneness? We hypothesized that the levels of shame proneness would be reduced over the course of ICBT treatment and that shame proneness would mediate the relationship between ICBT modules and social anxiety symptoms. If successful, this investigation will further elucidate the treatment of SAD and contribute new insights into the development of more detailed and targeted ICBT programs.

## Methods

### Study Design and Approval

This research was an 8-week clinical trial. Participants were recruited from 2015 to 2017 in two different stages: a pilot study, which consisted of only the self-help and the wait list control (WLC) groups, and a controlled trial with 3 groups. All data came from a larger program of ICBT. This study was approved by the local ethics committee and registered in Peking University. The trial registration number is ChiCTR1900021952.

### Participants and Eligibility Criteria

We used a community sample in the study. Participants were recruited through different internet platforms, and they were informed about the basic information, aim, and procedure of the study. Individuals who were interested in the study were required to finish several self-reported questionnaires on the website (N=1479). In addition, they were invited to participate in the Chinese version of the Mini International Neuropsychiatric Interview (MINI; N=784) [24,25]. The MINI was conducted either face-to-face or through telephone by 3 masters-level graduate students and 1 doctoral student in clinical psychology, all of whom have learned and practiced MINI under the guidance of a professional psychiatrist and have gained certain clinical interview skills.

**Figure 1:**
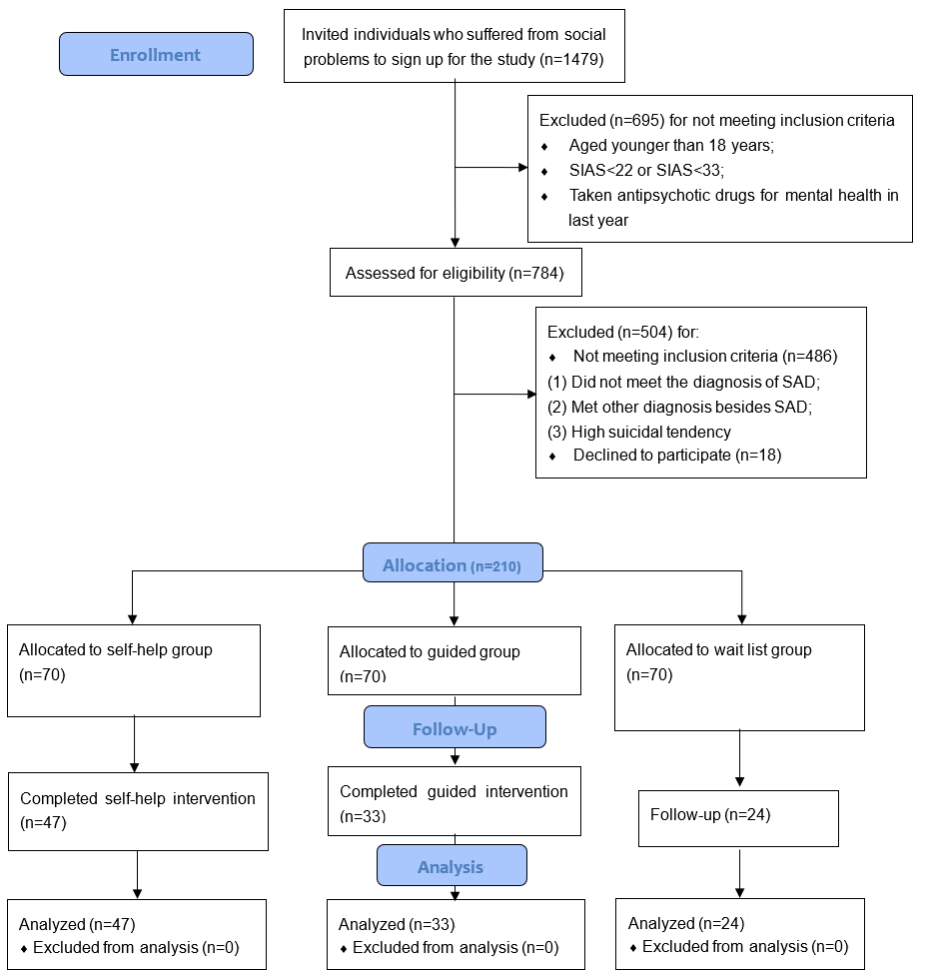
Flowchart of this study.

The main inclusion criteria were as follows: participants who were older than 18 years and who met the diagnostic criteria of SAD in the Structural Clinical Interview for Diagnostic and Statistical Manual of Mental Disorders, 4th edition (DSM-IV) Axis I Disorders. Their Social Interaction Anxiety Scale (SIAS) score was higher than 22, with Social Phobia Scale (SPS) score higher than 33. They did not take any antipsychotic drugs or undergo other psychological treatments in the last year, and they did not meet the diagnostic criteria of schizophrenia, bipolar disorder, and high suicidal tendency. Participants had to agree that they could finish the 8-week ICBT program and the posttreatment measurements. Detailed information of the screening process and the eligibility criteria are shown in Figure 1.

### Participants’ Basic Information

A total of 104 participants, including 31 males and 73 females, aged 18 to 45 years (mean 24.92, SD 4.59 years) met the criteria and agreed to attend the treatment program.

### Measures

#### Experience of Shame Scale

The 25-item Experience of Shame Scale was composed by Qian et al [26] and designed to measure participants’ shame proneness. Higher scores represent higher shame proneness. The scale has high reliability and validity (standard Cronbach alpha=.87).

#### Social Interaction Anxiety Scale and Social Phobia Scale

SIAS is a 19-item scale, originally composed by Mattick and Clarke and revised into a Chinese version [27,28]. SIAS is used to evaluate the degree of individuals’ feeling of anxiety and fear in a social interaction situation, such as being in a party or talking to others. SPS is another scale that assesses the anxiety and avoidance when individuals are being observed by others in social situations [28]. These two scales are often used together, and they both have high internal reliability (0.87 for SIAS and 0.90 for SPS) and retest reliability (0.86 for SIAS and 0.85 for SPS). The criterion-related validity of SIAS is 0.514 and of SPS is 0.479.

#### Beck Depression Inventory

The Chinese version of the Beck Depression Inventory (BDI) scale is widely used in the measurement of depressive symptoms, with high reliability (standard Cronbach alpha=.890 and split-half reliability=0.879) and validity [29]. Prior research showed that the relationship between SAD and depression is high [30]; thus, to eliminate the influence of depressive symptoms, the BDI score and the result of MINI were both seen as excluding criteria for the screening process.

#### Mini International Neuropsychiatric Interview

The MINI [25] is a structured interview with high internal reliability (0.94) and test-retest reliability (0.97), designed to access participants’ psychotic symptoms according to DSM-IV, revised. The interview takes approximately 30 min.

The primary outcome measures were changes in the ESS score and the relationships of ESS and ICBT, whereas others were recorded as secondary outcomes.

### The Internet-Based Cognitive Behavioral Therapy Program

The ICBT program is an internet-based self-help cognitive behavioral intervention course and was first developed at the University of Bern [6]. The original materials were translated and revised twice by 9 clinical psychologists from the School of Psychological and Cognitive Sciences at Peking University. Except for some course practices that were modified because of cultural differences, no other contents were changed.

The 8-week courses can roughly be divided into 5 parts. First, motivation arousing, which guides the participants to think about and write down why they want to change and what life would be like if social anxiety symptoms reduce. Relaxation training would also be introduced to participants in this module. Second, psychoeducation, which explains the relevant theories of SAD, the concepts of negative thoughts, safety behaviors, self-focus attention, and their relationships, helping participants gradually construct the case formulation of their own. Third, cognitive construct, which instructs participants to identify and re-examine their nonadaptive negative thoughts and to take notes on the *rational thinking* form, which will guide them to replace nonadaptive thoughts with adaptive ones. Fourth, attention training, which helps participants to focus more on the external environment other than themselves. Fifth, exposure and problem solving, which aim to help participants to confront the situations that may cause anxiety, to try behavioral experiments, and to solve problems.

Overall, two forms of the ICBT intervention were included in the study: the self-help ICBT and guided ICBT. A total of 3 therapists were included in the program, all of whom were masters-level graduate students in clinical psychology, who had undergone formal CBT training and had at least 1 year of experience of individual counseling, and they were supervised by a licensed clinical psychologist on a weekly basis. Each therapist assists a certain number of participants when needed in the guided group. The assistance of the therapists consisted of a weekly email to each patient, aiming at motivating and reinforcing their usage of the ICBT program. Furthermore, therapists answered participants’ questions about the ICBT program. Therapists also needed to know the basic information of their patients and their progress in the program, the last time of their visit, and the homework record. Approximately 15 min were needed to prepare and reply to the email per patient for each week. The program had an independent network platform for therapists, and they can check the login information and relative data of all participants on the platform (such as their homework and the time they spent on each module).

### Information Collecting and Research Process

After the screening process, the participants would first sign the digital informed consent form via internet and were provided with the instructions of the program. After which they would be divided into 3 groups: guided group, self-help group, and WLC group. Each individual needed to fill out the SPS, SIAS, and ESS scales before and immediately after completing the ICBT program (or 2 months later for the WLC group).

### Statistical Analysis

All analyses were conducted using SPSS version 20 (IBM Corp). First, differences among various groups in demographic and pretreatment clinical variables were tested using chi-square and one-way analysis of variance (ANOVA) tests. Repeated measures of ANOVA were also conducted to verify the participants’ improvement after ICBT.

For further analysis, we introduced a variable, residual gain (RG), to indicate the intervention changes. A linear regression model was fitted to find the specific modules of ICBT, which have an impact on RG of shame proneness. Afterward, we conducted a mediation analysis to investigate the role of shame proneness in the ICBT treatment. The results related to ESS (shame proneness) were regarded as the primary outcome.

## Results

### Participants

The descriptive statistics of all variable scores are shown in Table 1. In total, participants’ mean age was 24.92 (SD 4.59) years, and 70.2% (73/104) of them were female. Using pretreatment scores of SPS, SIAS, and ESS as dependent variables, we conducted three 2 (gender) × 3 (group) univariate ANOVA. The results showed did not yield significant main effects of group (SIAS: *F*_2,98_=1.532; *P*=.22; partial η²=0.03; SPS: *F*_2,98_=1.034; *P*=.359; partial η²=0.021; and ESS: *F*_2,98_=0.257; *P*=.77; partial η²=0.005) and gender (SIAS: *F*_1,98_=0.102; *P*=.75; partial η²=0.001; SPS: *F*_1,98_=0.084; *P*=.77; partial η²=0.001; and ESS: *F*_1,98_=0.257; *P*=.77; partial η²=0.005), and the interaction effects were also not significant (SIAS: *F*_2,98_=0.489; *P*=.615; partial η²=0.01; SPS: *F*_2,98_=1.571; *P*=.21; partial η²=0.031; and ESS: *F*_2,98_=0.176; *P*=.839; partial η²=0.004). This suggested that the 3 groups of participants had the same level of shame and social anxiety before ICBT.

**Table 1:** The descriptive statistics of all variables before and after the treatment.

Sociodemographics		Intervention (n=80)									*F* (df)		*P* value		Chi-square (*df*=2)
		Self-help (n=47)			Guided (n=33)			Wait list (n=24)							
		Values	*d*	Values		*d*	Values		*d*						
**Gender,** **n (%)**															0.9
	Female	34 (72)	N/A	22 (67)		N/A	17 (71)		N/A	N/A		N/A			
Age (years), mean (SD)		25.91 (4.25)	N/A	24.73 (5.40)		N/A	23.25 (3.59)		N/A	2.81 (2,101)		.07			
**Education level, n (%)**															0.9
	Low/middle	31 (66)	N/A	22 (67)		N/A	16 (67)		N/A	N/A		N/A			
	High	16 (34)	N/A	11 (33)		N/A	8 (33)		N/A	N/A		N/A			
**Diagnosis, n (%)**															0.9
	SAD^b^	23 (49)	N/A	15 (45)		N/A	9 (38)		N/A	N/A		N/A			
	SAD+MDD^c^	9 (19)	N/A	9 (27)		N/A	6 (25)		N/A	N/A		N/A			
	SAD+AD^d^	8 (17)	N/A	5 (15)		N/A	5 (20)		N/A	N/A		N/A			
	SAD+MDD+AD	7 (15)	N/A	4 (12)		N/A	4 (16)		N/A	N/A		N/A			
**Experience of Shame Scale** **, mean (SD)**		N/A	0.80	N/A		0.88	N/A		0.25	N/A		N/A		N/A	
	Pretreatment	75.45 (10.53)	N/A	74.61 (12.94)		N/A	76.29 (12.48)		N/A	0.14 (2,101)		.87			
	Posttreatment	68.11 (13.92)	N/A	64.64 (14.97)		N/A	76.92 (13.13)		N/A	5.47 (2,101)		.006			
**Social Interaction Anxiety Scale, mean (SD)**		N/A	1.01	N/A		1.20	N/A		0.21	N/A		N/A		N/A	
	Pretreatment	66.62 (10.62)	N/A	70.67 (9.36)		N/A	66.04 (10.61)		N/A	1.96 (2,101)		.15			
	Posttreatment	53.91 (14.80)	N/A	51.21 (13.25)		N/A	66.50 (13.11)		N/A	9.27 (2,101)		<.001			
**Social Phobia Scale** **, mean (SD)**		N/A	0.89	N/A		0.96	N/A		0.25	N/A		N/A		N/A	
	Pretreatment	56.02 (14.16)	N/A	53.48 (13.87)		N/A	55.42 (13.74)		N/A	0.33 (2,101)		.72			
	Posttreatment	44.89 (17.54)	N/A	40.03 (14.99)		N/A	56.29 (16.90)		N/A	6.83 (2,101)		.002			

^a^N/A: not applicable.

^b^SAD: social anxiety disorder.

^c^MDD: major depressive disorder.

^d^AD: other anxiety disorders.

### Dropout Rate and Adherence

The dropout rate difference between the self-help (32.86%) and guided (52.86%) ICBT groups was significant, with higher dropout rate in the guided group (χ²_1_=5.7; *P*=.02). In addition, we identified another two adherence indexes, the number of modules and homework finished in the ICBT program. A moderation analysis was conducted to investigate whether shame proneness moderated the relationship between ICBT form and treatment adherence. The results did not yield any significant effect (for the module number: β=−.0193; SE 0.0374; 95% CI −0.0938 to 0.0551 and for the homework number: β=−.0393; SE 0.0406; 95% CI −0.1202 to 0.0415). In this regard, shame proneness is not a moderator in the relationship between the form of ICBT and treatment adherence.

### Primary Outcomes

We used repeated measures of ANOVA to access whether ICBT can reduce participants’ shame proneness. The results showed that the interaction effect of group and time on ESS (*F*_2,135_=8.44; *P*<.001; partial η²=0.11) was significant. Simple effect analysis showed that after treatment, the ESS scores of intervention groups were significantly reduced (self-help: mean deviation [MD]=7.34; Cohen *d*=0.80; 95% CI 3.99 to 10.69; *P*<.001 and guided: MD=9.97; Cohen *d*=0.88; 95% CI 5.36 to 14.58; *P*<.001). As a result, ICBT was effective for the reduction of both social anxiety symptoms (SIAS and SPS) and shame proneness (ESS).

#### The Analysis of Effect of Internet-Based Cognitive Behavioral Therapy on Shame Proneness

In our study, RG (post-pre) was used as an improvement index of the ICBT treatment. RG is calculated as follows: Z2−(Z1×r12), in which Z2 means the Z score of posttreatment, Z1 is the pretreatment Z score, and r12 refers to the Pearson correlation of pre- and posttreatment scores [31]. The greater the absolute value is, the more the participant improves. Compared with the difference between pre- and posttest, the RG is not correlated with the pretest score, therefore eliminating the interference of the irrelevant variable.

To investigate the relationship between different levels of shame and ICBT, we analyzed the Pearson correlation between the pretest ESS score (ESS-pre) and the RG of social anxiety (RG-SIAS and RG-SPS). The results did not show significant correlations (RG-SIAS: *r*=−0.002; *P*=.98 and RG-SPS: *r*=0.12; *P*=.31). This result showed that ICBT had the same effect among participants with different shame levels.

Thus, we further investigated which module of ICBT had an effect on the decrease of shame. Using the feedback system of the network platform, we analyzed the Pearson correlation between each module’s involvement (using frequency and time as the indexes) and pretest ESS score and RG-ESS (the RG of ESS). The results showed that there was no significant correlation between pretest ESS and any involvement index; however, the frequency of relaxation training (*r*=−0.24 [.01<*P*<.05]; *P*=.03), the total number of words in graded exposure (*r*=−0.23 [.01<*P*<.05]; *P*=.04), the average number of words of graded exposure (*r*=−0.36 [*P≤*.01]; *P*=.001), and the average number of words of systematic problem solving (*r*=−0.25 [.01<*P*<.05]; *P*=.03) all had significant correlations with RG-ESS. In another words, there were no differences among participants with different degrees of shame proneness on their initiative preference of treatment tasks, but the more they involved in the relaxation training, problem solving, and exposure modules of ICBT, the more they improved on their shame level.

Furthermore, we used ENTERING method to perform a linear regression analysis on gender, age, group, the frequency of relaxation training, the total number of words writing in graded exposure and the average number of words writing in graded exposure as well as systematic problems. This linear regression analysis determined whether these parameters had influenced the score of RG-ESS. The results showed that the goodness-of-fit was the highest when the regression model included only gender and the average number of words of graded exposure as the predictive variables (*R*²_adjusted_=0.147; *F*_1,77_=9.79; *P*=.002). The regression coefficients (β) and the corresponding tests of significance are presented in Table 2. These results showed that both gender and the average number of words of exposure had a significant influence on the decrease in shame proneness: women improved more than men and the more the number of words of exposure, the more reduction in their shame level. Furthermore, we also calculated the Pearson correlation between the average number of words of exposure and participants’ depressive symptoms and found no significant correlation (with pretest BDI: *r*=0.09; *P*=0.44 and with posttest BDI: *r*=−0.15; *P*=.20), indicating that depressive symptoms did not show an impact on the involvement of exposure module and its effect on shame proneness.

**Table 2:** The regression coefficients of the regression model.

Variable	β	SE	*t* value (*df*=2,77)	*P* value	Tolerance	Variance inflation factor
Constant	N/A^a^	N/A	1.10	.27	N/A	N/A
Gender	.21	0.175	1.97	.05	0.98	1.02
The average number of words of graded exposure	−.33	0.002	−3.13	.002	0.98	1.02

^a^N/A: not applicable.

#### The Mediation Effect of the Change of Shame Level

**Figure 2:**
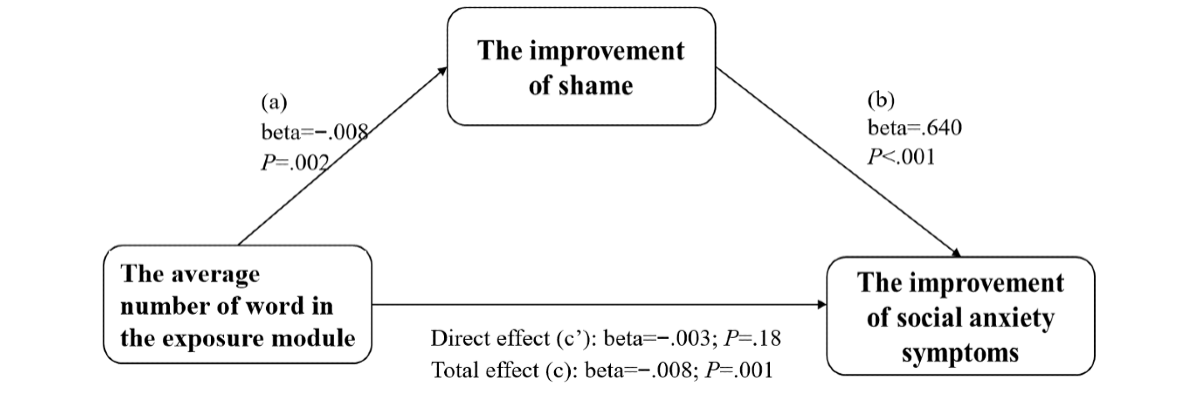
The mediation effect of shame decrease on exposure to Social Interaction Anxiety Scale.

**Figure 3:**
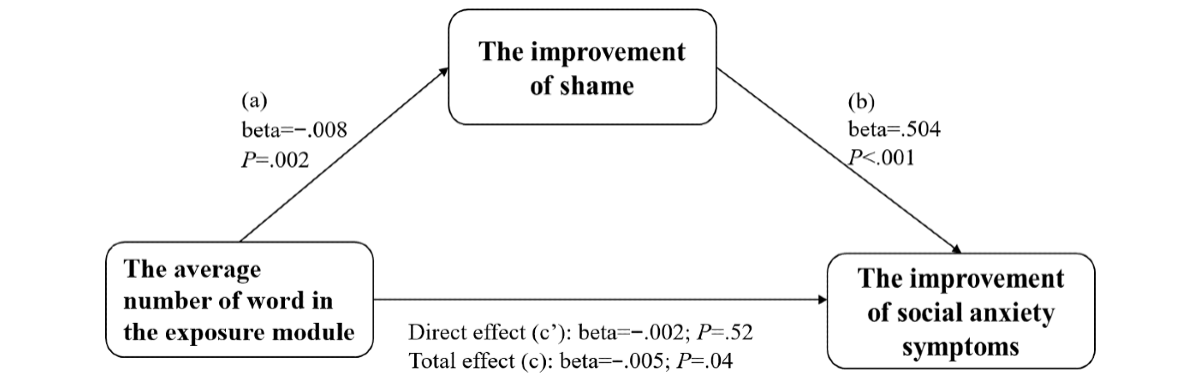
The mediation effect of shame improvement on exposure to Social Phobia Scale.

To further investigate the relationship among ICBT, shame proneness, and social anxiety, we did a mediation analysis. We used the average number of words of the exposure module as the predictive variable, the RG of ESS (the decrease of shame) as the mediation variable, and the RG of SIAS and SPS (the improvement of social anxiety) as dependent variables. The results of our analysis revealed evidence of a significant indirect effect of the average number of words of the exposure module on the improvement of social anxiety symptoms via their decrease of shame (for SIAS: β=−.0049; SE 0.0016; 95% CI −0.0085 to −0.0019 and for SPS: β=−.0039; SE 0.0015; 95% CI −0.0075 to −0.0012). Two graphical depictions of the model were seen in Figures 2 and 3, along with the statistics measuring the significance of each predictive pathway. Consistent with the results, the average number of words in the exposure module significantly predicted the RG of ESS (a path: β=−.0076; SE 0.0022; t_78_=−3.52; *P*<.001). In addition, the higher the improvement of shame, the higher the improvement of social anxiety symptoms (b path; for SIAS: β=.6397; SE 0.1366; t_78_=4.68; *P*<.001 and for SPS: β=.5037; SE 0.1380; t_78_=3.65; *P*<.001). Furthermore, the direct effect of the number of words in exposure to social anxiety improvement after controlling for the mediating influence of the decrease in shame proneness (RG-ESS) was not significant (c’ path; for SIAS: β=−.0030; SE 0.0022; t_78_=−1.34; *P*=.18 and for SPS: β=−.0015; SE 0.0023; t_78_=−0.65; *P*=.52). The mediation effects were still significant even if depressive symptoms were controlled. These results suggest that the decrease of shame level fully mediates the improvement of social anxiety symptoms (CI does not include 0).

### Secondary Outcomes

#### Changes in Social Anxiety and Their Level of Shame After Internet-Based Cognitive Behavioral Therapy

We conducted a group (self-help, guided, and WLC) × time (pre-/posttreatment) repeated measures of ANOVA analysis to access the treatment effect of ICBT. The results showed that the interaction effects of group and time on SIAS (*F*_2,101_=18.59; *P*<.001; partial η²=0.27) and SPS (*F*_2,101_=7.91; *P*=.001; partial η²=0.14) were significant. Simple effect analysis showed that the post-SIAS and post-SPS scores were significantly lower than pretreatment in both the self-help (SIAS: t_46_=7.06; Cohen *d*=1.01; 95% CI 9.08 to 16.32; *P*<.001 and SPS: t_46_=4.41; Cohen *d*=0.89; 95% CI 3.99 to 10.69; *P*<.001) and guided groups (SIAS: t_32_=8.28; Cohen *d*=1.20; 95% CI 14.67 to 24.24; *P*<.001 and SPS: t_32_=5.28; Cohen *d*=0.96; 95% CI 8.26 to 18.64; *P*<.001) but not in the WLC group.

#### The Influence of Two Treatment Groups on Treatment Effects

We used the RG of SIAS, SPS, and ESS as dependent variables and conducted independent *t* analysis to explore the group differences of treatment effect. The results showed that there was a marginal significant difference between SIAS RGs of the two groups (t_78_=1.88; *P*=.06); guided group showed more improvement than the self-help group. In addition, the RGs of two groups’ ESS (t_78_=1.09; *P*=.28) and SPS (t_78_=1.07; *P*=.29) had no significant difference. In other words, the SIAS score of the guided group was improved than that of the self-help group, but there was no difference between the two groups’ changes of shame level.

## Discussion

### Principal Findings and Interpretation

Our study used the Chinese version of the ICBT program to investigate whether shame can be significantly reduced during the treatment of SAD and which modules of ICBT exert an influence on the decrease of shame proneness. This study found significant reductions in participants’ shame proneness and social anxiety scores over the course of ICBT treatment in both the self-help and guided groups. Our study also suggested that gender (being female) and level of involvement in the exposure module (ie, higher average word count in completed homework assignments) were the only two significant predictors of reductions in shame proneness.

Furthermore, shame proneness fully mediated the relationship between the participants’ average word count in the exposure module and change in social anxiety scores. That is, greater engagement in the exposure module led to greater improvements in shame proneness, which, in turn, led to greater improvements in social anxiety symptoms.

As for the dropout rate and adherence, our results showed that the dropout rate of the guided group was significantly higher than that of the self-help ICBT group, and shame proneness was not a moderator in the relationship between the form of ICBT and treatment adherence.

### Comparison With Prior Work

The effectiveness of both the self-help and guided ICBT on SAD showed in this study is consistent with previous research [6,32-34]. Primary and secondary outcome measures showed significant changes and moderate to large treatment effects after the ICBT program. This is comparable with the treatment effects reported in a recent study [34]. Furthermore, our results indicated that shame played a role in this process.

The relationship between shame and social anxiety has long been debated. According to the psychoevolutionary model [13,35], individuals with social anxiety tend to excessively focus on their social rank and think of themselves in an inferior position, which causes a series of reactions, such as avoiding eye contact, blushing, and timidity [36]. Shame plays an important role in this process [37]. A longitudinal study demonstrated that a clinical group intervention that aimed at reducing shame-proneness could also reduce participants’ social anxiety symptoms. Li et al [38], indicated that shame proneness might be a risk factor of SAD. Neuroimaging studies also support this theory. Using structural magnetic resonance imaging scans, Syal et al [39] found that the gray matter of the frontal, temporal, parietal, and insular cortices of the right hemisphere of pateints with SAD was thinner than those of controls. Particularly, thinner anterior cingulate cortex and posterior cingulate cortex (PCC) thickness were associated with higher levels of shame proneness [40] as well as higher severity of social anxiety symptoms [39,41]. According to prior studies, PCC is considered to be involved in the process of social cognition [42] and re-experiencing of past events [43], which are both essential to the maintenance of shame proneness.

In addition, our study indicated that shame proneness played a mediation role in the relationship between the participants’ average word count in the exposure module of the ICBT and change in social anxiety scores. Some previous evidence might explain the mediational model. Many studies have confirmed that early negative experiences (such as emotional neglect and abuse) have an influence on feelings of shame and social anxiety, which are subsequently internalized, causing more stable shameful-based schemas [44,45]. To support this view, Fung and Alden [46] demonstrated that being rejected in social situations exerted an influence on the subsequent development of social anxiety. Other researchers further proposed and verified the following path: early negative experience causes shame proneness, which predicts a coping strategy of self-criticism to hide one’s perceived defects and prevent the shameful situation from re-emerging, which eventually develops into social anxiety symptoms [47]. Together, these findings indicate that early negative experiences, which are usually treated using exposure therapy, might be important factors in the etiology of shame proneness and social anxiety. As such, it is not surprising that numerous studies have suggested that shame proneness can be alleviated through exposure [48-50] and that shame proneness mediates the relationship between the interruption of avoidant behaviors and reductions in social anxiety.

Furthermore, our study showed a higher dropout rate in the guided ICBT group compared with the self-help group. This might be because of more perceived burden of participants in the guided group, who thought of the email support as another homework. Haug et al [51] offered an explanation that the mature ICBT program has already included the motivation enhancement and psychoeducation, which are the main aims of the therapists’ guidance. Therefore, the guidance is not necessarily helpful to the intervention. The relationship between the guided ICBT and adherence is mixed in previous studies [52,53]. This inconsistency might be related to the different forms and time length of the guidance. In addition, two more progressive adherence variables were identified in our research, and we found that shame proneness was not a moderator in the relationship between the form of ICBT and treatment adherence. Our results indicated that adding more contact with the therapist did not have an impact on adherence and homework completion. A possible explanation is that the email guidance, essentially internet based, may be too short in time to develop a good therapeutic alliance, which is an important factor to provide reinforcement for adherence. Therefore, the additional improvement of therapeutic contact could not be taken place via email.

### Limitations

There are several limitations worth noting in this study. First, our study did not investigate the follow-up effect of ICBT on shame proneness and social anxiety symptoms. Future research is needed to explore the long-term effects of ICBT on these constructs and their interaction. Second, it remains possible that the sequence of the interventions may have contributed to the mediation effect. ICBT is a continuous therapy with 8 different modules, in which exposure is the last one. Participants’ motivation and involvement of the exposure might be influenced by previous modules, which we were not able to differentiate in this study. Finally, in our study, the exposure was implemented as a one-time intervention, whereas the measurement of shame proneness was measured as change over the course of the 8-week intervention, which may also confound the effects of other aspects of the intervention. Future dismantling studies are needed to separate these influences and further verify this mediation effect by using only the exposure intervention rather than the entire ICBT package.

### Conclusions

In accordance with the theories mentioned earlier, our results suggest that shame proneness is an important factor in treating SAD and can be reduced through engagement in a web-based, self-guided exposure treatment. To our knowledge, this is the first study to investigate the mediation effect of shame proneness in the relationship between ICBT (particularly the exposure component of ICBT) and social anxiety symptoms. Our results suggest that among all the ICBT modules we investigated, only the completion of the exposure component significantly improved social anxiety symptoms by reducing the level of shame proneness. In short, this investigation further elucidates a process-based approach to alleviate shame and social anxiety and contribute insights into the development of more tailored exposure-based ICBT programs.

## Abbreviations

ANOVA: analysis of variance
BDI: Beck Depression Inventory
CBT: cognitive behavioral therapy
DSM-IV: Diagnostic and Statistical Manual of Mental Disorders, 4th edition
ESS: Experience of Shame Scale
ICBT: internet-based cognitive behavioral therapy
MD: mean deviation
MINI: Mini International Neuropsychiatric Interview
PCC: posterior cingulate cortex
RG: residual gain
SAD: social anxiety disorder
SIAS: Social Interaction Anxiety Scale
SPS: Social Phobia Scale
WLC: wait list control
